# Sexual-Sparing Radical Cystectomy in the Robot-Assisted Era: A Review on Functional and Oncological Outcomes

**DOI:** 10.3390/cancers17010110

**Published:** 2025-01-01

**Authors:** Carlo Introini, Manfredi Bruno Sequi, Marco Ennas, Andrea Benelli, Giovanni Guano, Antonio Luigi Pastore, Antonio Carbone

**Affiliations:** 1 E.O. Ospedali Galliera, UOC Urologia, 16128 Genova, Italy; 2 Urology Unit, Department of Medico-Surgical Sciences & Biotechnologies, Faculty of Pharmacy & Medicine, Sapienza University of Rome, 04100 Latina, Italy

**Keywords:** bladder cancer, sexual-sparing, urinary function, sexual function, outcomes, radical cystectomy, robotic, nerve-sparing

## Abstract

**Background/Objectives**: Radical cystectomy (RC) is the standard treatment for muscle-invasive and high-risk non-muscle-invasive bladder cancer, but it often results in significant functional impairments, including sexual and urinary dysfunction, adversely affecting quality of life (QoL). Sexual-sparing robotic-assisted radical cystectomy (RARC) has been introduced to mitigate these effects. This review evaluates the oncological and functional outcomes of sexual-sparing RARC in male and female patients. **Methods**: A systematic literature search identified 15 studies including 793 patients who underwent sexual-sparing RARC using techniques such as nerve-sparing, capsule-sparing, and pelvic organ-preserving approaches. Data on oncological and functional outcomes were analyzed. **Results**: Sexual-sparing RARC achieves oncological outcomes comparable to open RC, with negative surgical margin (NSM) rates exceeding 95% in most studies. RFS and CSS rates were robust, often surpassing 85% at intermediate follow-ups. Functional outcomes were also favorable, with continence rates exceeding 90% and erectile function recovery surpassing 70% in well-selected male patients. Female patients undergoing pelvic organ-preserving techniques demonstrated improved continence, preserved sexual function, and enhanced QoL. Patient selection emerged as critical, favoring those with organ-confined disease and good baseline function. **Conclusions**: Sexual-sparing RARC offers a promising balance between oncological control and functional preservation, making it an effective option for selected patients. Further research is needed to refine techniques and establish standardized protocols for broader adoption.

## Simple Summary

Bladder cancer is among the most common malignancies worldwide, often requiring radical cystectomy (RC) for muscle-invasive and high-risk non-muscle-invasive cases. While effective, this procedure frequently leads to significant functional impairments, including urinary incontinence and sexual dysfunction, adversely affecting quality of life. In response, sexual-sparing techniques in robot-assisted radical cystectomy (RARC) have emerged as a promising approach to improve functional outcomes without compromising oncological control. This review examines the latest evidence on sexual-sparing RARC, highlighting its potential to preserve sexual and urinary function in both male and female patients. Techniques such as nerve-sparing, capsule-sparing, and pelvic organ-preserving approaches show encouraging functional outcomes. In select patients, oncological outcomes align closely with those of standard RC. Careful patient selection remains crucial, favoring those with organ-confined disease and good baseline function. While early data is promising, further prospective studies and standardized protocols are needed to validate these findings and facilitate broader clinical adoption. Sexual-sparing RARC represents a step forward in balancing cancer control with improved postoperative quality of life.

## 1. Introduction

Bladder cancer (BC) represents the tenth most common cancer globally, with incidence rates particularly high among the male population, ranking as the sixth most common cancer in this group. The highest rates were observed in developed countries [1]. BC is linked to numerous risk factors, yet many patients are diagnosed without any evident exposures [2]. Cigarette smoking is the most significant contributor to the rising incidence of bladder cancer in Western countries. Furthermore, the severity of smoking may influence the aggressiveness of the disease, as heavy smokers have a higher likelihood of developing high-risk non-muscle invasive BC (NMIBC) and muscle-invasive BC (MIBC) [3]. Radical cystectomy (RC) with pelvic lymph node dissection and urinary diversion (UD), with or without neoadjuvant chemotherapy to date, represents the “gold standard” in the treatment for MIBC, for high- and very high-risk NMIBC [4]. However, this procedure is associated with a high complication rate, significantly impacting patients’ quality of life (QoL) [5]. Moreover, RC can adversely affect urinary, bowel, and sexual function (SF), as well as body image, leading to a decline in QoL, thus potentially causing psychological distress [6,7]. While the construction of an orthotopic neobladder (ON) offers the benefit of preserving body image, postoperative complications such as urinary incontinence (UI)—particularly nocturnal urinary incontinence (NUI)—can significantly impact the QoL [8]. Regarding SF, erectile dysfunction (ED) rates range from 20% to 100% [9,10,11], while loss of sexual desire, orgasm disorders, dyspareunia, and vaginal lubrication disorders are commonly reported in female patients [12]. Concerns about impaired functional outcomes are important factors influencing the decision-making process for both urologists and patients, particularly in younger individuals [13]. For this reason, efforts to preserve sexual function after surgery have led to the development of specialized techniques that are designed to reduce the impact on patients’ QoL [9]. Over the years, this approach has been refined through multiple variations, each aiming to optimize outcomes for voiding and SF in patients undergoing RC. To date, the European Association of Urology recognizes four different sexual-sparing techniques in males: prostate-sparing, capsule-sparing, seminal-sparing, and nerve-sparing approaches [4]. In females, pelvic organ-preserving techniques involve preserving the neurovascular bundles, vagina, uterus, ovaries, or variations of any of the stated techniques [13]. Technological improvement has aided in the development of minimally invasive surgical approaches such as laparoscopic RC (LRC) and then robot-assisted RC (RARC) [14]. RARC has attracted substantial attention within the urologic community over the past few decades, significantly increasing its use due to its better ergonomics and shorter learning curve [15,16]. Moreover, RARC is linked to lower blood loss, faster postoperative recovery, and shorter hospitalization compared to ORC [17]. Such technical innovations have, thus, granted surgeons the opportunity to improve and further modify the above-mentioned sexual-sparing techniques. In this review, the term ‘sexual-sparing radical cystectomy’ encompasses a range of surgical techniques aimed at preserving sexual and urinary function by safeguarding neurovascular bundles, the endopelvic fascia, and reproductive organs such as the prostate, seminal vesicles, uterus, and vagina. This narrative review aims to explore the evolving landscape of sexual-sparing surgical techniques in radical cystectomy, with a particular focus on minimally invasive surgery (MIS), evaluating both functional and oncological outcomes.

## 2. Materials and Methods

Starting in August 2024, a comprehensive search across Pubmed and Scopus was conducted to identify relevant studies on minimally invasive sexual-sparing techniques in RC for both male and female patients. The search terms included “sexual-sparing radical cystectomy”, “robot-assisted sexual-sparing cystectomy”, “nerve-sparing cystectomy”, and “organ-sparing radical cystectomy”. The search was limited to English-language studies involving human subjects published between 2000 and 2024.

Studies were excluded for reasons such as focusing on simple cystectomy, lack of a clear surgical approach definition, descriptive surgical technique reports without outcome data, or being case reports. After screening, 15 studies with a total of 793 patients met the inclusion criteria.

A narrative review was chosen to broadly synthesize existing evidence, accommodating variability in study designs and patient populations across the available literature. This approach allows for a comprehensive overview without the strict methodological constraints of a systematic review.

## 3. Evidence Synthesis

A total of 15 studies recruiting a total of 793 patients (including both intervention and control groups [Table 1]) were included in this review, comprising 4 comparative studies (all retrospective non-randomized comparative studies [NRCSs]) and 11 single-arm case series [18,19,20,21,22,23,24,25,26,27,28,29,30,31,32]. The comparative studies included in this review addressed three primary types of comparisons: nerve-sparing (NS) versus non-nerve-sparing (NNS) RC [18,21,22,23; capsule-sparing versus standard RC [19]; and organ-sparing versus non-organ-sparing RC [20]. Most of the studies reviewed focused on patients receiving ONB reconstruction; however, a small proportion of patients received ileal conduit or continent cutaneous diversion.

### 3.1. Urinary Continence

Urinary continence (UC) outcomes following RARC with ON reconstruction have consistently demonstrated favorable results in both male and female patients, with the success of recovery heavily influenced by the preservation of critical anatomical structures. Maintaining the neurovascular bundles, pelvic floor integrity, and autonomic pathways is essential for achieving high rates of daytime and nighttime continence in men. Similarly, in women, the preservation of the vaginal wall, pelvic floor, and autonomic nerve pathways is paramount for effective reconstruction of continence mechanisms.

#### 3.1.1. Urinary Continence in Male Patients

Daytime continence (DC) was rapidly and universally achieved in most studies, often 100% by 12 months, with some cohorts demonstrating earlier recovery. For instance, nerve-sparing and partial seminal-sparing RARC reported 75% DC within one month, progressing to 100% by 12 months [24]. Similarly, prostate apex-preserving RARC, which maintains membranous urethral length and stability of the urethral–neobladder anastomosis, achieved 100% DC within the first year [25]. Supporting these findings, Jonsson et al. [26] observed a 97% DC rate at 12 months, emphasizing the efficacy of nerve-sparing techniques. Advanced approaches such as intra-fascial nerve-sparing further accelerated recovery, with patients achieving 100% DC by six months due to meticulous preservation of autonomic and somatic innervation [18]. The detrusor apron-sparing technique also reached 100% DC at 12 months by maintaining the structural integrity of the lower detrusor apron and perivesical tissues [27]. Capsule-sparing techniques and anterior suspension further enhanced outcomes by minimizing damage to neurovascular structures and providing stability to the neobladder–urethral anastomosis. He et al. [19] demonstrated 94.7% DC rates at six months and 96.7% nighttime continence (NC) rates at 12 months with these combined approaches, underscoring the importance of structural stability and nerve preservation. NC recovery, although slower, showed promising improvements with techniques prioritizing autonomic nerve preservation. Autonomic pathways, which are critical for involuntary sphincter control, require longer recovery periods. Studies reported 72.5% NC at 12 months following nerve-sparing RARC [24], while apex-preserving approaches yielded outcomes of 33.3% of complete NC [25]. Jonsson et al. [26] reported 83% NC at 12 months in patients undergoing their neobladder reconstruction, and intra-fascial NS techniques achieved rates at 12 months of 63.6% [18]. However, detrusor apron-sparing approaches achieved 64% NC at 12 months, suggesting that factors like bladder compliance and individual recovery trajectories influence outcomes [27]. Notably, exceptional early continence recovery was observed with endopelvic fascia-sparing RARC. In this cohort, 100% achieved DC and 77.7% achieved NC within 30 days, which was attributed to the preservation of the pelvic floor and autonomic pathways’ structural integrity [28]. These findings highlight the critical role of surgical precision and anatomical preservation in optimizing both voluntary and autonomic continence recovery.

#### 3.1.2. Urinary Continence in Female Patients

**Figure 1:**
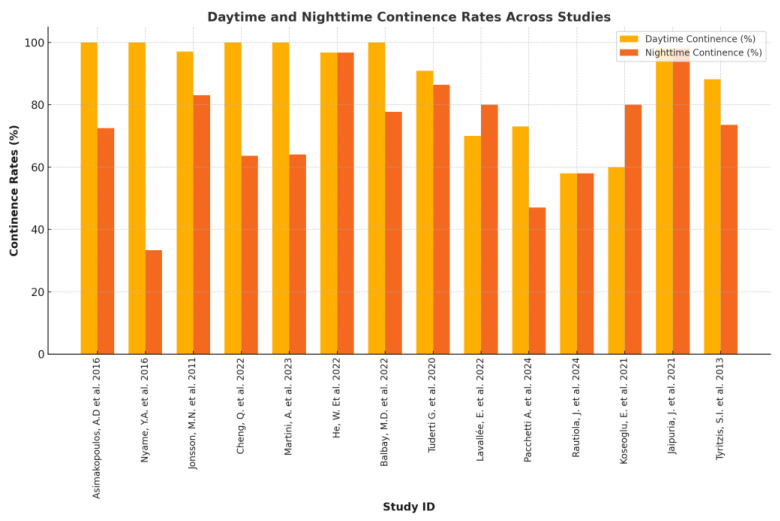
Graphical representation of continence outcomes [18,19,20,21,22,24,25,26,27,28,29,30,31,32].

DC rates across studies ranged from 70% to 90.9% at 12 months, underscoring the critical importance of preserving pelvic structures during pelvic organ-preserving and sex-sparing RARC. Key anatomical structures, such as the vaginal wall, pelvic floor, and autonomic nerve pathways, are essential for supporting the reconstructed neobladder and urethral sphincter, making their preservation pivotal for continence recovery. Sex-sparing approaches demonstrated the most favorable outcomes, with 90.9% of women achieving DC within 12 months due to meticulous pelvic anatomy preservation [29]. Lavallée, in their pelvic organ-preserving RARC series, reported a 70% DC rate at one year through maintaining neurovascular bundles and autonomic pathways vital for sphincter function [30]. Anterior vagina wall preservation techniques demonstrated DC rates of up to 73% [31]. Rautiola et al. further confirmed that careful dissection and autonomic nerve preservation significantly improve continence outcomes [20]. Outcomes varied in smaller studies based on surgical technique and patient-specific factors. For instance, in a genitalia-preserving cohort, some patients achieved full continence, while others experienced varying degrees of incontinence, influenced by preoperative bladder function, surgical complexity, and nerve preservation [32]. NC recovery, which is generally more challenging than daytime, improved significantly when autonomic nerve pathways were preserved. Sex-sparing surgery yielded 86.4% NC recovery at 12 months [29], and pelvic organ-preserving RARC achieved 83% over the same period [30]. In contrast, non-preserving approaches struggled, with only 47% attaining nocturnal control [31]. Variability was also seen in genitalia-preserving RARC, with outcomes ranging from good to poor depending on individual factors [32] such as adherence to rehabilitation protocols, age and comorbidities. Overall, these findings highlight the clear advantages of pelvic organ-preserving and sex-sparing techniques in optimizing both daytime and nighttime continence. Maintaining the pelvic anatomy’s structural and functional integrity remains key, but outcome variability emphasizes the need for refined surgical strategies and personalized postoperative care. Early rehabilitation and targeted interventions, such as pelvic floor muscle training, are crucial to closing the gap between daytime and nighttime control, ultimately improving patients’ QoL after RARC. Figure 1 shows a graphical representation of continence outcomes.

#### 3.1.3. Clean Intermittent Self-Catheterization (CISC)

CISC rates in male patients were below 5% in the studies examined. This is attributed to the anatomical simplicity of the male urethra and surgical techniques that preserve pelvic and autonomic structures. Jaipuria et al. reported a CISC rate of less than 1% with the Pitcher Pot neobladder modification, which optimizes bladder compliance and urethral alignment [21]. By contrast, CISC rates in women range from 20% to 50%, reflecting the greater complexity of reconstructing continence mechanisms. Incomplete voiding, often due to reduced bladder compliance or pelvic floor dysfunction, is the primary indication. For instance, Pacchetti et al. and Tuderti et al. reported 26.3% and 27.2 CISC rates, respectively [29,31], and Lavallée reported a 50% CISC rate [30]. These findings underscore the need for optimized neobladder design and postoperative interventions tailored to the female anatomy. Table 2 summarizes urinary functional outcomes.

### 3.2. Sexual Function

#### 3.2.1. Sexual Function in Male Patients

Erectile function (EF) recovery after NS RARC depends heavily on preserving the neurovascular bundles and adjacent pelvic structures that are essential for penile erections. These anatomical features near the prostatic fascia contain somatic and autonomic nerve fibers that control erectile function. The precision and meticulous execution of NS techniques, particularly intra-fascial approaches, are critical for optimal outcomes. Intra-fascial NS techniques consistently outperform inter-fascial approaches, which preserve finer neurovascular fibers closer to the prostatic fascia. For instance, patients undergoing intra-fascial NS RARC achieved a 100% potency rate (IIEF > 20) at 12 months, compared to 50% in inter-fascial cases [18]. Robotic-assisted surgery enhances the precision of these delicate dissections, minimizing mechanical and thermal damage to the neurovascular bundles. In one cohort, 77.5% of patients regained EF within 3 months, while 72.5% achieved preoperative IIEF scores by 12 months, demonstrating the impact of robotic technology in facilitating nerve and seminal sparing [24]. Structural preservation, particularly of the endopelvic fascia, also plays a pivotal role in EF recovery. Fascia-sparing techniques, which maintain the integrity of autonomic pathways, enable rapid functional recovery. In one study, 85.7% of preoperatively potent patients maintained normal erectile function (IIEF-5 ≥ 19) within 30 days [28]. Similarly, bilateral NS consistently yields better outcomes than unilateral or non-sparing approaches, with 81.2% potency recovery reported in one analysis [22]. Patient-specific factors, such as age and preoperative erectile function, significantly influence recovery. Younger patients and those with robust baseline potency are more likely to regain EF postoperatively. For example, 88% of preoperatively potent patients maintained EF after surgery, emphasizing the importance of patient selection and counseling in setting realistic expectations [23]. Even in advanced cases requiring more aggressive dissection, preserving as much anatomical integrity as possible enhances recovery while maintaining oncological safety. Pharmacological support serves as an important adjunct to surgical techniques in EF recovery. Approximately 55% of patients benefit from PDE5 inhibitors to achieve functional erections, while others may require additional interventions, such as vacuum erection devices or intracavernosal injections [27]. These tools, combined with precise NS surgery and targeted rehabilitation, underscore the multi-faceted approach required to restore erectile function. Despite the inherent challenges, including advanced disease or large tumor burdens, techniques such as intra-fascial NS, fascia preservation, and robotic precision consistently deliver excellent EF outcomes. These findings highlight the importance of tailoring surgical strategies to each patient’s anatomical and clinical needs, striking a balance between functional recovery and oncological safety.

#### 3.2.2. Sexual Function in Female Patients

Sexual function is an essential aspect of postoperative QoL in women undergoing pelvic organ-preserving and sex-sparing RARC. Preserving neurovascular bundles, the vaginal wall, and pelvic autonomic pathways is critical for maintaining lubrication, arousal, orgasm, and sexual activity. While data remain limited compared to male outcomes, studies highlight the benefits of tailored surgical techniques. Tuderti et al. reported that 72.7% of women resumed sexual activity within one year, with improvements in arousal, lubrication, and orgasm scores compared with the 3 month mark [29], while Lavallée et al. found a similar recovery in 87% of patients [30]. These outcomes underscore the importance of preserving vaginal and neurovascular integrity for optimal recovery. However, challenges persist, as Pacchetti et al. noted that Female Sexual Function Index (FSFI) scores remained below preoperative levels, suggesting the need for additional interventions such as sexual counseling and therapy [31]. Genitalia-preserving RARC, as demonstrated by Koseoglu et al. [32], provides another promising approach by preserving the uterus, ovaries, and vaginal wall, though detailed outcomes on satisfaction require further study. Beyond physical recovery, psychological factors, including body image and emotional well-being, play a vital role in resuming sexual activity. Despite challenges, advancements in sex-sparing techniques and comprehensive postoperative care offer meaningful recovery opportunities. Table 3 summarizes the findings on sexual function.

### 3.3. Oncological Outcomes

**Figure 2:**
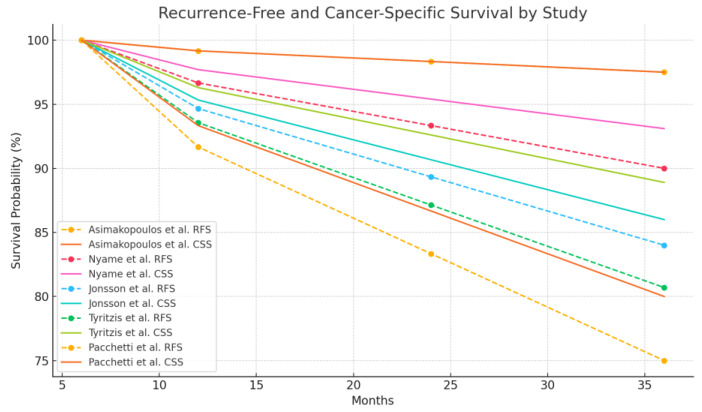
Graphical representation of oncological outcomes [22,24,25,26,31].

RARC has demonstrated oncological outcomes comparable to open radical RC (ORC) [14]. Key metrics—including negative surgical margins (NSM), recurrence-free survival (RFS), cancer-specific survival (CSS), and lymph node dissection (LND) yields—suggest that RARC can be an effective surgical option for bladder cancer. However, the variability in results, particularly in advanced disease stages, highlights the importance of cautious interpretation and appropriate patient selection. High NSM rates are consistently reported in studies of RARC. Asimakopoulos et al. [24] observed a 97.5% NSM rate in their cohort, with a single case of a soft tissue-positive margin in a pT3a tumor. Preservation-focused approaches, including fascia-sparing [28] and detrusor apron-sparing [27], also achieved NSM rates of 100%. These results suggest that RARC can achieve high surgical precision. Still, positive margins in advanced cases, such as those reported by Koseoglu et al. (40% positive margins in pT4 cases) [32], underscore the challenges in more aggressive diseases. RFS and CSS further reflect the oncological reliability of RARC. Asimakopoulos et al. reported a 97.5% RFS at 26.5 months, and Jonsson et al. observed 84% RFS and 86% CSS at 36 months. Tyritzis et al., evaluating bilateral NS RARC, noted an RFS of 80.7% at 30 months, suggesting that nerve-preserving approaches do not necessarily compromise cancer control [22,24,26,29]. In female patients undergoing organ-preserving RARC, studies by Tuderti et al. and Lavallée et al. reported 100% and 83% RFS, respectively [29,30]. However, Pacchetti et al. documented lower CSS at 80% over three years, with distant metastases in 27.3% of patients, emphasizing the need for improved strategies in high-risk and advanced cases [31] (Figure 2).

Lymph node dissection (LND) in RARC consistently meets oncological standards, with yields comparable to ORC. Balbay et al. reported a mean yield of 33.2 nodes [28], while Martini et al. and Jonsson et al. [26,27] achieved yields of 24.5 and 19 nodes, respectively. Even in capsule-sparing approaches, He et al. demonstrated comparable lymph node retrieval, suggesting that functional preservation does not necessarily compromise staging accuracy [19]. However, the variability in yields across studies highlights the potential influence of surgical expertise and technique. Advanced disease poses challenges to RARC, with mixed outcomes reported in studies involving patients with higher-stage tumors. Nyame et al. observed no local recurrences in their prostate apex-preserving cohort, though one nodal recurrence occurred at 31 months and was managed with chemotherapy [25]. In contrast, Pacchetti et al. noted higher rates of distant metastases, underscoring the limitations of RARC in managing advanced disease without systemic therapy [31]. Neoadjuvant chemotherapy (NAC) is frequently utilized in high-risk patients to enhance oncological outcomes. Studies by Asimakopoulos, Tyritzis, and Pacchetti reported NAC administration in 70–90% of cases, contributing to effective tumor downstaging [22,24,31]. Despite these benefits, the reliance on NAC highlights the need for a multimodal treatment approach, particularly in patients with aggressive or advanced disease. Table 4 summarizes the oncological outcomes, and Table 5 summarizes the comparative studies’ findings.

## 4. Discussion

Since its introduction, robot-assisted surgery has gained widespread popularity despite the lack of evidence on its superiority over open or laparoscopic procedures [33,34]. RC has seen a different kind of evolution over the years, with more gradual improvements being made due to the intrinsic morbidity of the procedure [35,36]. Several trials have been conducted comparing RARC with ORC [37,38]. The iROC trial, in particular [37], showed lower readmission rates (21.8% vs. 32.2%), fewer non-cancer-related deaths (0.6% vs. 1.9%), reduced wound complications (5.6% vs. 17.3%), and a lower incidence of venous thromboembolism (1.9% vs. 8.3%), thus positioning RARC with intracorporeal diversion as a gold-standard approach alongside ORC. Regardless of the surgical approach, RC with ONB diversion has a high prevalence of sexual and urinary dysfunction [9], significantly impacting the patient’s QoL. Various surgical techniques have been developed and implemented to mitigate these effects over the years [11,39,40,41]; however, until recently, the field of sexual-preserving RARC was almost unexplored. In reviewing the literature, the throughline that we found was that the success of sexual-sparing RARC depends on precise patient selection that balances oncological safety with functional preservation. This approach is most effective in patients with organ-confined disease (≤pT2), where high negative surgical margin rates and excellent cancer-specific survival have been consistently reported. Studies such as those by Tuderti et al. and Asimakopoulos et al. highlight how the precision of robotic systems allows for nerve- and organ-sparing techniques without compromising oncological outcomes [24,29]. However, the retrospective nature of most studies introduces limitations, including selection bias, incomplete data, and confounding factors, leading to overestimating functional outcomes while underreporting complications. Future prospective multicentric randomized trials are necessary to reinforce the current body of evidence and validate these findings. Patients with advanced-stage tumors, such as pT3–T4 or multifocal disease, present significant challenges. For example, Koseoglu et al. demonstrated a 40% positive margin rate in patients with pT4 tumors, underscoring the limitations of sexual-sparing techniques in managing aggressive disease [32]. In such cases, oncological control must take precedence over functional preservation. Lymph node involvement also plays a critical role in determining suitability for sexual-sparing RARC. Comprehensive lymph node dissection is essential for accurate staging and local control, and the median lymph node yields reported in sexual-sparing RARC studies (18–33 nodes) are comparable to those in open cystectomy [26,27,28]. However, patients with extensive nodal disease may require broader dissections, which can conflict with nerve preservation efforts. Additionally, the presence of aggressive histological variants, such as squamous or small-cell carcinoma, often precludes functional preservation techniques due to their high recurrence rates and poor prognosis [24]. Functional outcomes, particularly continence and sexual function, are equally critical in evaluating candidates. Patients with strong baseline function stand to gain the most from nerve-sparing and organ-preserving techniques. Haberman et al. demonstrated that preoperatively potent patients undergoing bilateral nerve-sparing RARC experienced significant recovery of erectile function postoperatively [23]. However, patients with severe baseline dysfunction may derive limited benefit, and prioritizing oncological goals may be more appropriate. Younger, healthier patients are better candidates for sexual-sparing approaches due to their ability to tolerate longer operative times and recover more effectively. Older or frailer patients may achieve better outcomes with more straightforward surgical techniques. Aligning these decisions with patient preferences and expectations is paramount. Some patients may prioritize preserving sexual and urinary function over oncological risks, while others may favor complete disease control above all else. Preoperative counseling ensures that surgical planning aligns with the patient’s values and goals. The technical feasibility of sexual-sparing RARC also depends on anatomical considerations and prior interventions. Factors such as pelvic anatomy, prior radiation, or pelvic surgery can complicate nerve preservation. Failure to select appropriate candidates can lead to suboptimal outcomes, including compromised oncological safety, higher rates of recurrence, and diminished functional benefits. These outcomes emphasize the necessity of balancing oncological rigor with functional goals and highlight the importance of patient-specific surgical planning. To summarize, sexual-preserving techniques should be reserved for favorable candidates with organ-confined disease (≤pT2), no bladder neck or urethral involvement, and good baseline urinary and sexual function. Furthermore, younger patients and those with localized tumors were consistently identified as ideal candidates due to their greater capacity for functional recovery and improved cancer control. Several limitations have been found in the studies examined. Among the studies included in the review, long-term follow-up data remain scarce, thereby limiting insights into the durability of both functional and oncological outcomes. While the intermediate-term results seem encouraging, future studies must prioritize extended follow-up to assess the sustainability of sexual function and continence over time, while maintaining good oncological outcomes. Furthermore, the representation of female patients accounted for less than 15% of total participants. Despite promising outcomes from pelvic organ-preserving approaches, the generalizability of these findings is constrained due to the gender imbalance. Female-specific studies have shown that pelvic organ-preserving techniques in female patients, such as vaginal wall and uterine preservation, demonstrated improved functional outcomes, with daytime continence rates of up to 90% and preserved sexual function. Preserving neurovascular bundles and autonomic pathways is essential in minimizing postoperative complications, ultimately enhancing QoL. This review highlights the potential of sex-preserving RARC. However, future research should prioritize randomized controlled trials to provide higher-quality evidence and larger studies focusing on female patients to explore gender-specific outcomes. Investigation of nocturnal incontinence and erectile function with longer follow-up durations, standardization of surgical techniques, and rehabilitation protocols should be carried out to optimize functional preservation without compromising oncological outcomes.

## 5. Conclusions

In conclusion, sexual-sparing RARC represents a technically feasible procedure by offering a promising balance between oncological safety and functional preservation, making it an effective option for well-selected patients with organ-confined disease. High rates of negative surgical margins, favorable survival outcomes, and improved quality of life underscore its potential. However, careful patient selection and further research into long-term outcomes and accessibility remain essential. With continued advancements, sexual-sparing RARC may become a cornerstone in bladder cancer management, particularly for patients prioritizing functional preservation. Larger cohorts, randomized controlled trials, and multi-institutional efforts have to be implemented in order to allow these surgical techniques to become more widely used.

## Figures and Tables

**Table 1:** Baseline characteristics of studies included.

Study ID	Type of Study	Enrolment Period	Type of Surgery	Urinary Diversion	Total Patients
Asimakopoulos, A.D et al., 2016 [24]	Retrospective single-arm case series	2011–2014	Nerve/seminal-sparing	Orthotopic Neobladder	40
Nyame, Y.A. et al., 2016 [25]	Prospective single-arm case series	2013–2014	Apex-sparing	Orthotopic Neobladder	3
Jonsson, M.N. et al., 2011 [26]	Prospective single-arm case series	2004–2009	Nerve-sparing	Orthotopic Neobladder (36), Ileal conduit (9)	45
Cheng, Q. et al., 2022 [18]	Retrospective Comparative	2018–2019	Intrafascial nerve-sparing vs. interfascial nerve-sparing vs. non-nerve-sparing	Orthotopic Neobladder	33
Martini, A. et al., 2023 [27]	Retrospective single-arm case series	2017–2021	Lower detrusor apron-sparing	Orthotopic Neobladder	11
He, W. Et al. 2022 [19]	Retrospective Comparative	2017–2021	Standard RARC vs. Lateral capsule-sparing + combined pelvic reconstruction vs. combined pelvic reconstruction	Orthotopic Neobladder	107
Balbay, M.D. et al., 2022 [28]	Retrospective single-arm case series	2019–2022	Endopelvic fascia-sparing	Orthotopic Neobladder	10
Tuderti G. et al., 2020 [29]	Retrospective single-arm case series	2013–2018	Sexual organ-sparing	Orthotopic Neobladder	11
Lavallée, E. et al., 2022 [30]	Retrospective single-arm case series	2008–2020	Pelvic organ-sparing	Orthotopic Neobladder (20), Ileal conduit (3)	23
Pacchetti A. et al., 2024 [31]	Retrospective single-arm case series	2017–2022	Anterior vaginal wall preservation/total preservation if no bladder neck, urethra or posterior wall infiltration	Orthotopic Neobladder	22
Rautiola, J. et al., 2024 [20]	Retrospective Comparative	Not specified	Organ-sparing vs. non organ-sparing	Orthotopic Neobladder	146
Koseoglu, E. et al., 2021 [32]	Prospective single-arm case series	Not specified	Genitalia-sparing	Orthotopic Neobladder	5
Jaipuria, J. et al., 2021 [21]	Retrospective case series	2007–2017	Nerve-sparing vs. Non-nerve-sparing	Orthotopic Neobladder	238
Tyritzis, S.I. et al., 2013 [22]	Retrospective Comparative	2003–2012	Nerve-sparing vs. Non-nerve-sparing	Orthotopic Neobladder	70
Haberman, K. et al., 2014 [23]	Retrospective single-arm case series	2003–2012	Nerve-sparing vs. Non-nerve-sparing	Orthotopic Neobladder (26), Indiana Pouch (2), Ileal conduit (1)	29

**Table 2:** Principle urinary function outcomes.

Study ID	Measurement	Daytime (%)	Nighttime (%)	CISC (%)
Asimakopoulos, A.D et al., 2016 [24]	<1 pad/day	100, 12 mo	72.5, 12 mo	0
Nyame, Y.A. et al., 2016 [25]	<1 pad/day	100, 12 mo	33.3, 12 mo	0
Jonsson, M.N. et al., 2011 [26]	≤1 pad/day	97, 12 mo	83, 12 mo	NA
Cheng, Q. et al., 2022 [18]	≤1 pad/day	100, 12 mo	63.6, 12 mo	0
Martini, A. et al., 2023 [27]	≤1 pad/day	100, 12 mo	64, 12 mo	NA
He, W. et al., 2022 [19]	≤1 pad/day	96.7, 12 mo	96.7, 12 mo	NA
Balbay, M.D. et al., 2022 [28]	<1 pad/day/Safety pad	100, 1 mo	77.7, 1 mo	NA
Tuderti G. et al., 2020 [29]	Self-report	90.9, 12 mo	86.4, 12 mo	27.2
Lavallée, E. et al., 2022 [30]	≤1 pad/day/Safety pad	70, 12 mo	80, 12 mo	50
Pacchetti A. et al., 2024 [31]	≤1 pad/day	73, 12 mo	47, 12 mo	26.3
Rautiola, J. et al., 2024 [20]	≤1 pad/day	58, 12 mo	58, 12 mo	NA
Koseoglu, E. et al., 2021 [32]	≤2 pad/day	60, 3 mo	80, 3 mo	0
Jaipuria, J. et al., 2021 [21]	≤1 pad/day	98, 15 mo	98, 15 mo	<1
Tyritzis, S.I. et al., 2013 [22]	≤1 pad/day	88.2, 12 mo	73.5, 12 mo	1 patient

**Table 3:** Principle sexual function outcomes.

Study ID	Measurement	EF	Sexual Activity	Treatment
Asimakopoulos, A.D et al., 2016 [24]	IIEF ≥ 17	100%, 12 mo: 72.5% Return to preop IIEF scores	/	PDE5i
Nyame, Y.A. et al., 2016 [25]	Sexual Health Inventory for Men (SHIM)	100% SHIM ≥ 23	/	/
Jonsson, M.N. et al., 2011 [26]	IIEF ≥ 17	80% 12 mo	All but one patient had intercourse with or without PDEi at 3 mo	PED5i
Cheng, Q. et al., 2022 [18]	IIEF ≥ 20	100% intra-fascial NS 50% inter-fascial NS	/	/
Martini, A. et al., 2023 [27]	Self-reported	36% spontaneous erection 55% erection with PDE5i 9% intracavernous injection	/	PDE5i intracavernous injection
Balbay, M.D. et al., 2022 [28]	IIEF ≥ 19	85.7%, 3 mo	/	PED5i
Tuderti G. et al., 2020 [29]	FSFI	NA	72.7% were sexually active at 12 months	/
Lavallée, E. et al., 2022 [30]	Sexual function questionnaire	NA	87% were sexually active: Satisfaction rates: 38% high 54% moderate 8% low	/
Pacchetti A. et al., 2024 [31]	FSFI	NA	47% were sexually active Satisfaction rates: 72% high	26.3
Koseoglu, E. et al., 2021 [32]	Self-reported	NA	Inactive	/
Jaipuria, J. et al., 2021 [21]	Self -reported Question item 56 of BCI	“fair”, 15 mo	/	/
Tyritzis, S.I. et al., 2013 [22]	Self-reported	81.2%, 12 mo	50% of females sexually active	PDE5i intracavernous injection
Haberman, K. et al., 2014 [23]	Self-reported	88%, 12 mo	/	/

**Table 4:** Oncological outcomes.

Study ID	Tumor Stage	Median Follow-Up (Months)	NAC (%)	Positive Margins (%)	RFS (%)	CSS (%)	OS (%)
Asimakopoulos, A.D et al., 2016 [24]	T2–T3	26.5	15	2.5	97.5	97.5	97.5
Nyame, Y.A. et al., 2016 [25]	≤T2	28.2	33.3	0	67.7	67.7	67.7
Jonsson, M.N. et al., 2011 [26]	T0–T4	25	2.2	2.2	84	86	86
Cheng, Q. et al., 2022 [18]	T2–T3	17	12.1	3.1	100	100	100
Martini, A. et al., 2023 [27]	T0–T4	12	82	0	/	100	100
He, W. et al., 2022 [19]	T0–T4	12	12.1	2.3	/	/	/
Balbay, M.D. et al., 2022 [28]	T0–T4	11.5	10	0	90	100	100
Rautiola, J. et al., 2024 [20]	T0–T4	12	49	5	/	/	/
Tuderti G. et al., 2020 [29]	T0–T3	28	36.3	0	100	100	100
Lavallée, E. et al., 2022 [30]	T0–T4	20	57	4	83	91	91
Pacchetti A. et al., 2024 [31]	T0–T3	24	77	0	68.2	80	80
Koseoglu, E. et al., 2021 [32]	T1–T4	10	/	40	/	/	/
Jaipuria, J. et al., 2021 [21]	T1–T3	49	12.6	/	/	/	/
Tyritzis, S.I. et al., 2013 [22]	T0–T4	30.3	24.3	1.5	80.7	88.9	88.9
Haberman, K. et al., 2014 [23]	T0–(≥T2)	32.9	6.9	0	86.2	93.1	93.1

**Table 5:** Comparative studies’ findings.

Study ID	Control	Study	Control	Study	Control	Study
	Continence		Sexual Function		Oncological Outcomes	
Cheng, Q. et al., 2022 [18]	≤1 pad/day		IIEF ≥ 20		Incidental prosate cancer in one case 100% RFS	3.1% Positive surgical margins 100% RFS
	Daytime 90.9% Nighttime 0%	Daytime 100% Nighttime 63.6%	Not reported	100% Intra-fascial NS 50% Inter-fascial NS		
Rautiola, J. et al., 2024 [20]	≤1 pad/day		Not reported		Positive surgical margins: 5% cT ≥ 3: 33%	Positive surgical margins: 5% cT ≥ 3:13%
	Daytime 50% Nighttime 49%	Daytime 58% Nighttime 58%				
He, W. et al., 2022 [19]	≤1 pad/day		Not reported		Incidental prostate cancer: Standard: 13.6% Positive surgical margins Standard: 4.5	Incidental prostate cancer: LCS: 10% CPR: 11.6% Positive surgical margins: LCS: 0% CPR: 2.3%
	Standard Daytime 90% Nighttime 92.5%	Lateral capsule sparing Daytime 92.9% Nighttime 92.9% Combined pelvic reconstruction Daytime 96.7% Nighttime 96.7%				
Tyritzis, S.I. et al., 2013 [22]	≤1 pad/day		Self report		CSS: 81.2% Local recurrence: 4.8%.	CSS: 93.1% Local recurrence: 2.4%.
	Daytime 88.9% Nighttime 81.2%	Daytime 88.2% Nighttime 73.5% Pad-free: 27.5%	Potent without medication: 9.5% Potent with PDE5 inhibitors: 4.8% Not potent: 71.4%	Potent without medication: 31.2% Potent with PDE5 inhibitors: 50% Potent with intracavernosal injections: 3.1% Not potent: 6.2%		
